# Connected but at Risk: Social Media Exposure and Psychiatric and Psychological Outcomes in Youth

**DOI:** 10.3390/children12101322

**Published:** 2025-10-02

**Authors:** Giuseppe Marano, Francesco Maria Lisci, Sara Rossi, Ester Maria Marzo, Gianluca Boggio, Caterina Brisi, Gianandrea Traversi, Osvaldo Mazza, Roberto Pola, Eleonora Gaetani, Marianna Mazza

**Affiliations:** 1 Unit of Psychiatry, Fondazione Policlinico Universitario A. Gemelli IRCCS, 00168 Rome, Italymarianna.mazza@policlinicogemelli.it (M.M.); 2 Department of Neurosciences, Università Cattolica del Sacro Cuore, 00168 Rome, Italy; 3 Unit of Medical Genetics, Department of Laboratory Medicine, Ospedale Isola Tiberina-Gemelli Isola, 00186 Rome, Italy; gianandrea.traversi@gmail.com; 4 Spine Surgery Department, Bambino Gesù Children’s Hospital IRCCS, 00168 Rome, Italy; osvaldo.mazza1973@hotmail.it; 5 Section of Internal Medicine and Thromboembolic Diseases, Department of Internal Medicine, Fondazione Policlinico Universitario A. Gemelli IRCCS, Università Cattolica del Sacro Cuore, 00168 Rome, Italy; 6 Department of Translational Medicine and Surgery, Fondazione Policlinico Universitario A. Gemelli IRCCS, Università Cattolica del Sacro Cuore, 00168 Rome, Italy; eleonora.gaetani@unicatt.it; 7 Unit of Internal Medicine, Cristo Re Hospital, 00167 Rome, Italy

**Keywords:** adolescents, social media, mental health, anxiety, depression, body image, emotional regulation, suicidal ideation, neurodevelopment

## Abstract

**Highlights:**

**What are the main findings?**

- Passive and compulsive social media use in adolescents is associated with increased risks of depression, anxiety, body dissatisfaction, and suicidal ideation.
- Neurodevelopmental vulnerabilities, gender, emotion regulation difficulties, and problematic usage patterns act as key risk factors in shaping psychiatric outcomes.

**What are the implications of the main findings?**

- Social media is not inherently harmful; its psychological impact depends on usage patterns, individual traits, and social context.
- Prevention strategies should go beyond limiting screen time and focus on digital literacy, family engagement, emotion regulation, and platform responsibility.

**Abstract:**

**Background:** The widespread use of social media among children and adolescents has raised increasing concern about its potential impact on mental health. Given the unique neurodevelopmental vulnerabilities during adolescence, understanding how digital platforms influence psychiatric outcomes is critical. **Objectives:** This narrative review aims to synthesize current evidence on the relationship between social media exposure and key psychiatric symptoms in youth, including depression, anxiety, body image disturbances, suicidality, and emotional dysregulation. **Methods:** We conducted a comprehensive narrative review of the literature, drawing from longitudinal, cross-sectional, and neuroimaging studies published in peer-reviewed journals. Specific attention was given to moderators (e.g., age, gender, and personality traits) and mediators (e.g., sleep, emotion regulation, and family context) influencing the relationship between social media use and mental health outcomes. **Results**: Evidence indicates that certain patterns of social media use, especially passive or compulsive engagement, are associated with increased risk of depression, anxiety, body dissatisfaction, and suicidal ideation. Adolescent girls, younger users, and those with low self-esteem or poor emotional regulation are particularly vulnerable. Neuroimaging studies show that social media activates reward-related brain regions, which may reinforce problematic use. Family support and digital literacy appear to mitigate negative effects. **Conclusions:** Social media use is not uniformly harmful; its psychological impact depends on how, why, and by whom it is used. Multilevel prevention strategies, including media education, parental involvement, and responsible platform design, are essential to support healthy adolescent development in the digital age.

## 1. Introduction

**Figure 1:**
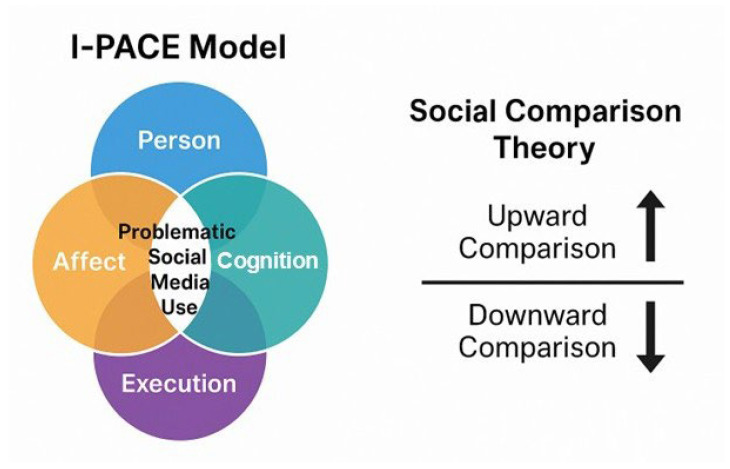
Conceptual frameworks explaining problematic social media use. This figure illustrates two complementary theoretical models. On the left, the I-PACE model (Interaction of Person-Affect-Cognition-Execution) conceptualizes problematic social media use as the result of dynamic interactions among individual traits, emotional states, cognitive responses, and executive control processes. On the right, Social Comparison Theory highlights the role of upward and downward comparisons in shaping users’ self-perceptions and emotional responses during social media engagement, which may contribute to maladaptive usage patterns.

Over the past decade, social media has become a dominant force in the daily lives of children and adolescents, fundamentally altering how young people interact, communicate, and construct their identities. Platforms such as Instagram, TikTok, Snapchat, and YouTube offer unprecedented opportunities for social engagement and self-expression. However, concerns have emerged regarding the potential psychological impact of frequent and early exposure to these digital environments [1,2,3]. Adolescence is a period of heightened neuroplasticity, emotional reactivity, and identity formation, making youth particularly sensitive to social feedback and peer comparison, which are core features of social media platforms [4,5]. Studies have reported associations between social media use and a range of mental health outcomes, including increased symptoms of depression, anxiety, body image dissatisfaction, emotional dysregulation, and suicidal ideation [6,7,8]. While some researchers argue that social media may foster social connection and peer support [9], others emphasize its role in promoting social comparison, cyberbullying, and addictive behavior patterns [10,11]. This divergence has led to ongoing debate in the literature. The question is if social media can be considered inherently harmful, or does its impact depend on how it is used and by whom. Several theoretical models, including the Interaction of Person-Affect-Cognition-Execution (I-PACE) model and Social Comparison Theory, have been proposed to explain these effects [12,13]. The I-PACE model is a theoretical framework developed to explain how and why individuals develop problematic or addictive behaviors in relation to internet and social media use. It stands for Interaction of Person, Affect, Cognition, and Execution. The model proposes that these components interact dynamically over time. A person with specific vulnerabilities (e.g., low self-esteem or high impulsivity) may experience negative emotions and use social media to cope. If the behavior is reinforced (e.g., relief, distraction, or positive feedback), it becomes habitual or compulsive. Weak executive functioning may impair the ability to disengage, leading to problematic or addictive use. The Social Comparison Theory explains how individuals evaluate their own abilities, appearance, and worth by comparing themselves to others. Social media platforms amplify the opportunity for social comparison, often under highly curated and unrealistic conditions. Adolescents, whose self-concept is still developing, are especially sensitive to these comparisons (Figure 1). 

Given the widespread use of social media among youth and the potential for long-term psychological consequences, there is a pressing need to clarify these associations and guide evidence-based prevention strategies. This narrative review aims to synthesize the existing literature on social media exposure and psychiatric outcomes in youth, with a focus on longitudinal evidence, neurodevelopmental mechanisms, and moderating factors such as age, gender, and family context. The review also highlights practical implications for clinical, educational, and policy domains, emphasizing that while social media use is not uniformly harmful, certain patterns of engagement may contribute to increased psychiatric vulnerability in young people. To avoid over-reliance on a small set of studies, we expanded coverage so that each key claim is supported by multiple sources across designs (large datasets, longitudinal/CLP, daily-diary/ESM, objective-log, and meta-analyses). We also include null/small effects where relevant to provide balance [14].

## 2. Materials and Methods

We conducted a narrative review aimed at synthesizing current evidence on the associations between social media exposure and psychiatric outcomes in youth. Given the breadth and heterogeneity of this literature (study designs, measures, and platforms), we prioritized a concept-driven synthesis over meta-analytic aggregation. We searched PubMed/MEDLINE, PsycINFO, Scopus, and Web of Science from January 2010 to August 2025, limited to English-language peer-reviewed publications. Reference lists of key papers were hand-searched to identify additional studies. Search strings combined terms for social media and youth mental health (e.g., “social media” AND adolescent* AND (depression OR anxiety OR suicide* OR “body image” OR “emotion regulation”)), with database-specific syntax. Regarding eligibility criteria, population was children and adolescents (≤19 years) or samples with separate adolescent analyses, exposure was social media use/engagement (active/passive/compulsive), platform-specific content, or objectively logged use, and outcomes were depressive and anxiety symptoms, suicidality/self-harm, body-image disturbance/disordered eating, emotion regulation/dysregulation.

Designs included cross-sectional, longitudinal, experience-sampling/daily diary, neuroimaging, and reviews/meta-analyses for theoretical/mechanistic context, while exclusions were editorials, non–peer-reviewed items, conference abstracts, case reports, and studies without relevant mental-health outcomes; non-English papers. Two reviewers independently screened titles/abstracts, then full texts, against eligibility criteria. Disagreements were resolved by consensus (third reviewer as arbiter when needed). Reasons for full-text exclusion were recorded. Two reviewers independently extracted study characteristics (country, sample, and design), exposure operationalization (time, activity type, and platform/content), outcome measures, and key estimates (effect sizes/CIs when reported). Findings were synthesized narratively, highlighting moderators (e.g., gender, age, and traits) and mediators (e.g., sleep, emotion regulation, and family context) and weighing evidence by design (longitudinal vs. cross-sectional) and typical sample sizes.

We extracted and reported effect sizes and 95% CIs where available, and we catalogued null findings (including objective-log and within-person designs) alongside positive associations. In the narrative synthesis, we weighed evidence by study design (cross-sectional vs. longitudinal vs. daily-diary/ESM) and measurement precision (self-report vs. objective logs). To improve interpretability, we report the design (cross-sectional, longitudinal, daily-diary/ESM, and neuroimaging), typical sample size, measurement source (self-report vs. objective logs), and any pre-registration notes for each key finding. When synthesizing, we give relatively greater weight to longitudinal and within-person (daily-diary/ESM) designs, studies using objective usage logs, and analyses with pre-registered protocols or robustness checks (e.g., specification curves). Cross-sectional associations are treated as non-causal and are primarily used to map correlational patterns. We also tracked directionality in longitudinal designs and, where available, report both paths (SMU → outcome and outcome → SMU), giving greater weight to estimates that establish temporal precedence and adjust for stable between-person differences [15,16].

Given the narrative scope, a formal tool-based risk-of-bias appraisal was not performed; nonetheless, we qualify conclusions by commenting on design limitations (self-report, inconsistent exposure definitions, and over-representation of cross-sectional designs) and by foregrounding longitudinal/objective-log studies where available.

A total of 1160 records were identified (databases n = 1142; registers n = 0; other sources/hand-search n = 18). After removal of duplicates (n = 362), 798 records were screened by title/abstract, and 608 were excluded. Full texts sought n = 190; not retrieved n = 9; full texts assessed for eligibility n = 181. Full-text exclusions n = 129 (wrong outcome/concept or context n = 58; wrong study design n = 44; non-adolescent sample or age not separable n = 27). In total, 52 studies were included in the review. Databases searched: PubMed/MEDLINE, PsycINFO, Scopus, Web of Science; search window: January 2010–August 2025.

## 3. Terminology and Measurement of Technology-Use Constructs

To avoid conflating distinct constructs, we adopt the following conventions. Problematic social media use (PSMU) denotes maladaptive engagement with social-networking platforms associated with impairment or risk; we use this label only for studies that operationalized PSMU with validated scales (e.g., Bergen Social Media Addiction Scale, BSMAS; Social Media Disorder scale, SMD). Problematic internet use (PIU), sometimes termed “internet addiction,” refers to generalized problematic engagement with the internet and is used here only when measured with instruments such as the Internet Addiction Test (IAT). Compulsive use (CU) indicates the compulsivity/loss-of-control dimension of online behavior and is used when assessed with the Compulsive Internet Use Scale (CIUS). Importantly, PSMU/PIU/CU are not formal DSM-5-TR or ICD-11 diagnoses and should not be equated with “addiction” unless diagnostic criteria are employed. Internet gaming disorder (IGD)/gaming disorder is reserved for gaming-specific behaviors (DSM-5-TR Section III; ICD-11) and is conceptually distinct from social-media use. Throughout the review, we report construct labels exactly as used in each study and, at first mention, provide the measurement instrument in parentheses (e.g., PSMU [BSMAS]; PIU [IAT]; CU [CIUS]). When authors used generic wording such as “social media addiction” without a validated measure, we describe the exposure as self-reported problematic use and interpret results cautiously [17,18,19,20].

## 4. Adolescent Brain Development and Vulnerability

Adolescence represents a critical period of neurodevelopment, characterized by significant structural and functional changes in the brain. During this stage, the limbic system, particularly the amygdala and ventral striatum, undergoes rapid maturation, enhancing emotional responsiveness and sensitivity to rewards. In contrast, the prefrontal cortex, responsible for executive functions such as inhibition, planning, and emotional regulation, develops more slowly, continuing into early adulthood [4,5]. This developmental mismatch may increase susceptibility to emotionally charged stimuli, such as those encountered on social media platforms.

Social media environments leverage this neurobiological sensitivity by providing immediate, high-frequency feedback through “likes,” comments, and shares. These features engage the brain’s dopaminergic reward systems, particularly the nucleus accumbens, which reinforces behaviors associated with social validation [21]. Adolescents, due to their heightened need for peer approval and incomplete self-regulation capacities, are especially prone to seek online affirmation in ways that may amplify anxiety and mood disturbances. Neuroimaging studies have demonstrated that adolescents exposed to high levels of social media engagement show increased activity in brain regions related to social cognition and emotional salience, including the anterior cingulate cortex and medial prefrontal cortex [22,23,24,25]. Such findings suggest that repeated digital social interactions may influence the trajectory of adolescent brain development in domains relevant to psychiatric functioning. In addition to neurobiological factors, adolescence is a pivotal time for identity formation and the consolidation of self-esteem. Exposure to highly curated online people and peer comparison may interfere with this process, increasing vulnerability to internalizing symptoms such as depression, anxiety, and disordered eating.

## 5. Types of Social Media Use

**Figure 2:**
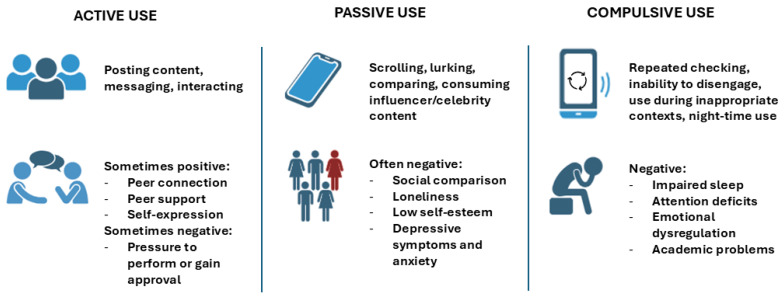
Types of social media use and associated psychological outcomes in adolescents.

The psychological impact of social media on adolescents is not determined solely by the amount of time spent online but rather by how, why, and in what contexts these platforms are used. Differentiating between usage patterns is essential to understanding the diverse ways in which social media may affect mental health (as shown in Figure 2).

A widely accepted distinction in the literature is between active and passive social media use. Active use involves intentional interaction with others: posting content, liking, commenting, and messaging. In contrast, passive use refers to browsing, scrolling, or viewing others’ posts without direct interaction. While active engagement can foster a sense of belonging and social connection, passive use has been consistently linked to negative emotional outcomes, including envy, loneliness, and reduced life satisfaction [26]. Passive users are more likely to engage in upward social comparison, viewing idealized portrayals of others’ lives that may distort reality and lower self-esteem. Several experimental studies suggest that even short periods of passive use can elevate negative affect, especially in adolescents prone to social sensitivity. Conversely, active use may buffer against these effects by facilitating social reciprocity and perceived social support, though the benefits appear modest and context-dependent [26].

The type of content consumed also plays a crucial role. Adolescents who engage primarily with appearance-focused or celebrity/influencer-driven content are more likely to experience body dissatisfaction and low self-worth [27]. Platforms like Instagram, Snapchat, and TikTok prioritize visual content, which is often filtered or edited to meet unrealistic beauty standards. The tendency to internalize these ideals is particularly strong among adolescent girls, who may use these platforms not only to consume but also to present curated versions of themselves. This dynamic contributes to the development of self-objectification, where individuals begin to view and evaluate their bodies from an external perspective [27]. Additionally, certain genres of content, such as fitness influencers, “what I eat in a day” videos, or before-and-after transformations, have been linked to the onset or worsening of disordered eating behaviors.

### Problematic and Compulsive Use

Although many studies use screen time as a proxy for exposure, the relationship between time spent and psychological distress is not straightforward. Some large-scale population studies have reported a dose–response relationship, where higher durations of daily use are associated with increased levels of depression and anxiety [28]. Other researchers have found that once basic needs for social interaction are met, additional time online has minimal additive impact [29]. Recent models advocate focusing on “screen quality” rather than “screen quantity.” For instance, interactions with close friends may improve well-being, while browsing strangers’ content without interaction may lead to isolation or negative social comparison. Additionally, night-time use may impair sleep, indirectly affecting mood and cognition. Thus, both timing (e.g., bedtime use) and motivations (e.g., seeking validation vs. boredom) are crucial variables.

A growing body of research addresses the phenomenon of problematic or compulsive social media use, sometimes conceptualized as a behavioral addiction. This pattern includes loss of control over usage, withdrawal-like symptoms when not connected, persistent checking even in inappropriate situations, negative impact on academic, social, or emotional functioning [30]. Problematic use is more prevalent among adolescents with existing vulnerabilities, such as low self-esteem, high impulsivity, emotional dysregulation, or social anxiety. These users may turn to social media as a maladaptive coping mechanism for stress, creating a vicious cycle of reinforcement. Some researchers have drawn parallels with substance use disorders, pointing to the involvement of reward-processing neural circuits and tolerance-like phenomena, where increasing engagement is required to achieve the same emotional effect [30]. Problematic or compulsive use of social media and the internet has been conceptualized by some researchers as a behavioral addiction. Individuals with internet addiction (IA) often display altered coping strategies, emotional dysregulation, and deficits in socio-emotional functioning, especially during adolescence, a developmental window marked by heightened vulnerability [31]. Neurobiological studies have revealed functional brain alterations in patients undergoing treatment for IA, including abnormal activation of craving-related brain circuits similar to those observed in substance use disorders [32]. These findings support the hypothesis that maladaptive digital behaviors may share common neurocognitive mechanisms with other addictive syndromes. Furthermore, IA has been examined in relation to pathological gambling, with evidence suggesting both overlapping and distinct psychopathological profiles. While both conditions are characterized by impaired impulse control and compulsivity, IA may present a different pattern of emotional regulation deficits and motivational drivers [33].

## 6. Psychiatric Outcomes Associated with Social Media Use

A growing body of research has explored how social media use affects the mental health of adolescents. Although not all forms of engagement are harmful, particular patterns of use have been consistently associated with adverse psychiatric outcomes, as summarized in Table 1. These effects tend to manifest most prominently as symptoms of depression, anxiety, body image disturbance, and suicidality. Rather than resulting from a single factor, these outcomes are shaped by the complex interplay of neurodevelopmental vulnerabilities, social dynamics, platform-specific features, and psychological predispositions. Consistent with our Methods, we refer to PSMU when studies used BSMAS/SMD, PIU when measured with IAT, and CU when measured with CIUS. We systematically report null and near-zero findings. Large multi-dataset specification-curve analyses show that digital technology use explains ≤ 0.4% of the variance (R^2^ ≤ 0.004) in adolescent well-being, indicating trivially small average associations [34]. Moreover, a recent daily-diary study with device-logged social media time found no consistent evidence for between-person or within-person links between objective screen-time and body-image dissatisfaction [35]. The presence of null or very small associations in high-quality designs (e.g., multi-dataset specification curves; objective-log daily diaries) suggests that average links between generic social media time and mental health outcomes may be weak and highly contingent on what is done online (content and context), who is using (individual vulnerabilities), and how outcomes are measured. Logged “time” appears to be a coarse proxy that often fails to capture risk-relevant mechanisms (e.g., upward social comparison, appearance-focused engagement, cybervictimization, and sleep disruption). Accordingly, we interpret pooled narrative signals with caution, highlight content/process-specific exposures, and give greater weight to longitudinal and within-person designs when drawing conclusions. We also explicitly acknowledge the potential for reverse causation and the role of measurement heterogeneity in producing contradictory results [35]. Most available evidence is cross-sectional, typically with samples in the hundreds to low tens of thousands, offering breadth but limited causal inference. Longitudinal cohorts (often n ≈ 500–10,000+) provide temporal ordering and, in several cases, identify mediated pathways (e.g., sleep, harassment, and self-esteem). Daily-diary/ESM studies (often n ≈ 100–500, many with device-logged use) clarify within-person dynamics and frequently report null or very small effects for generic screen-time metrics. Neuroimaging contributions (typically n ≈ 30–200) are mechanistic but underpowered for small effects. Across designs, measurement heterogeneity (exposure definitions and outcome scales) and reliance on self-report remain common limitations. Beyond study-level reporting, we considered recurring sources of bias when interpreting results: measurement source (self-report vs. objective logs), design (cross-sectional vs. longitudinal/CLP vs. daily-diary/ESM), analytic transparency (pre-registration/registered reports and robustness checks such as multiverse/specification-curve), sample size and attrition, and construct validity of exposures (time vs. content/process, e.g., appearance-focused engagement and cybervictimization). These considerations informed the narrative weighting (see Table 1 flags).

Across bidirectional longitudinal studies, depressive symptoms at baseline often predict later increases in problematic SMU, while SMU → later depressive symptom effects are inconsistent or null [16]. This pattern supports the importance of reverse-causation mechanisms alongside any potential causal effects of SMU on mental health [15].

### 6.1. Depression and Anxiety

Moderate but consistent associations between social media use and increased symptoms of depression and anxiety in adolescents have been described [26]. These relationships appear to be strongest when usage is passive or compulsive and when social media is used as a coping mechanism for distress. Adolescents who report high levels of social media engagement often experience disrupted sleep, greater sensitivity to peer feedback, and reduced face-to-face socialization. All these factors may contribute to internalizing symptoms. Gender differences have also been observed. Girls tend to use social media for relational purposes and are more likely to engage with appearance-focused content, which can increase vulnerability to mood disorders. The overwhelming message to adolescent girls is that their value is largely derived from their appearance, so girls tend to easily and frequently compare themselves to those they follow on Instagram, whether they are peers or celebrities [27]. Boys may be affected through different mechanisms, such as exposure to competitive or aggressive content, but current evidence suggests that girls consistently exhibit stronger correlations between social media use and depressive symptoms. Cohort evidence links higher social media/TV use to subsequent depressive symptoms (within-person) [41]. The UK cohort suggests indirect pathways via sleep, harassment, body image, and self-esteem (gender-differentiated) [37].

Importantly, the directionality of these effects is still debated. While longitudinal studies suggest that heavy social media use can precede increases in depression and anxiety, some adolescents may turn to digital platforms as a result of pre-existing mood disorders. In fact, some adolescents experiencing pre-existing mood disorders may turn to social media as a way to cope or seek support [42,43]. Depressed individuals are especially vulnerable to being involved in social media as they experience a stronger need to alleviate feelings of insecurity, low self-worth, or hopelessness. This bidirectionality underscores the need for nuanced interpretation and methodological rigor.

### 6.2. Body Image Disturbance and Disordered Eating

Adolescents, especially females, are often exposed to idealized beauty standards on social media platforms that rely heavily on visual content (e.g., Instagram and TikTok). Repeated exposure to these unrealistic and curated images may lead to dissatisfaction with one’s own appearance, a phenomenon that has been termed appearance-based social comparison [44]. The constant availability of filtered images, combined with public feedback mechanisms (likes and comments), encourages adolescents to internalize thin ideals and aesthetic perfection. Meta-analyses have confirmed that higher social media use is associated with increased body dissatisfaction, especially among users who engage in frequent comparison or who follow appearance-focused accounts [45]. Specifically, a small positive association overall, stronger for appearance-focused use has been described [46]; systematic reviews (e.g., Instagram) converge on content/process specificity; null daily-diary (objective logs) underscores that “time” is a coarse proxy [47]. Notably, the association between higher social media use and increased body dissatisfaction is not limited to girls. Boys, too, may internalize muscular or athletic ideals promoted through fitness influencers and visual advertising. These body image disturbances are not benign. They have been associated with the onset of disordered eating patterns, such as restrictive dieting, binge eating, and excessive exercise. Adolescents with high levels of body dissatisfaction are also more likely to report depressive symptoms, anxiety, and social withdrawal, suggesting a broader constellation of risk.

### 6.3. Suicidality and Self-Harm

One of the most concerning areas of research in adolescent psychiatry is the potential link between social media use and increased risk of suicidal ideation and non-suicidal self-injury (NSSI). While establishing a direct causal relationship remains challenging, multiple studies suggest that certain online environments may amplify pre-existing vulnerabilities, particularly in youth experiencing depression, trauma, or emotional dysregulation [48]. A key factor contributing to this risk is adolescents’ exposure to suicide-related content. On some platforms, users may encounter posts or discussions that normalize, romanticize, or even encourage self-harming behaviors. These can include so-called “suicide challenges,” hashtags related to self-injury, or graphic imagery that may act as a trigger for vulnerable individuals. This phenomenon, often described as suicide contagion, has been documented in offline contexts, such as schools or communities, and is now emerging in digital spaces as well. Another critical issue is the role of cyberbullying. Victims of online harassment often report intense feelings of rejection, humiliation, and hopelessness. Unlike traditional bullying, which is generally limited in time and space, cyberbullying can follow adolescents continuously due to the always-on nature of social media. In other words, digital harassment can occur at any time of day or night, without the boundaries of school hours or physical presence. This constant accessibility makes it difficult for victims to find respite or disengage from harmful interactions [44,45,48].

Furthermore, adolescents may use social media to disclose emotional distress or suicidal thoughts, but instead of receiving support, they may be met with indifference, ridicule, or negative feedback, potentially exacerbating their psychological state. The pressure to present a curated, emotionally restrained and idealized version of oneself may also discourage open dialogue about mental health struggles. These dynamics highlight how social media, when unmoderated or misused, can intensify emotional pain and compromise help-seeking behaviors in at-risk youth [49]. Not only young individuals may feel hesitant to share their vulnerabilities and challenges, fearing judgment or negative reactions, but this can lead to increased isolation and a reluctance to seek support for mental health issues.

## 7. Moderators and Mediators of the Relationship Between Social Media Use and Mental Health

The relationship between social media use and adolescent mental health is not uniform across all individuals. Instead, it is shaped by a constellation of moderating and mediating factors that influence either the strength of the relationship or the mechanisms through which it operates. Understanding these variables is essential to developing more accurate models and effective interventions.

The effects of social media vary significantly by age. Younger adolescents (e.g., ages 11–14), who are in early stages of identity formation and possess immature self-regulation capacities, tend to show higher vulnerability to online peer influence and social comparison. Longitudinal research suggests that the sensitivity to digital environments peaks during mid-adolescence, coinciding with a developmental window of heightened emotional reactivity and peer dependence [36]. Older adolescents may have more sophisticated cognitive strategies to buffer against negative effects but can still be affected by compulsive behaviors or addictive use patterns, particularly when social media is used as a maladaptive coping mechanism. Subjects presenting with such behaviors usually experience impulsivity, lack of emotional control, and, as a consequence, significant difficulties in managing conflicts.

As already outlined, gender plays a crucial moderating role in how adolescents experience social media. Girls are more likely to engage in appearance-based and relational social media use, which is strongly associated with depression, body dissatisfaction, and anxiety [50]. They also tend to be more active on platforms that emphasize self-presentation and peer approval (e.g., Instagram and TikTok). Boys, on the other hand, may gravitate toward content related to gaming, competition, or humor. While these themes may be less appearance-focused, boys may still experience problematic use, especially in the form of compulsive scrolling or late-night engagement, which can impair sleep and academic functioning.

Individual traits such as low self-esteem, high neuroticism, perfectionism, and high narcissistic traits have been found to moderate the psychological impact of social media [51]. Adolescents with these characteristics may be more prone to interpret ambiguous online interactions negatively, internalize social rejection, or seek validation through external metrics like likes and followers.

Moreover, emotion regulation abilities serve as a crucial mediator: adolescents who struggle to manage distress may turn to social media for distraction or emotional relief, potentially reinforcing a cycle of avoidance and emotional instability [52].

Contextual variables also play a mediating role. Adolescents with strong parental support, open communication in family, and positive offline relationships are more likely to experience social media as a tool for connection and exploration, rather than as a source of anxiety or self-doubt. Conversely, those with high levels of offline stress, such as family conflict, bullying, or academic pressure, may turn to social media as a form of escape. In such cases, digital engagement may worsen mental health by displacing protective offline activities like physical exercise, sleep, and face-to-face social interaction [53].

A clearer understanding of the social media–mental health link in adolescence requires distinguishing for whom and under what circumstances associations tend to emerge (moderators) from how and why they unfold (mediators). Read together, the evidence suggests that average effects look small when exposure is measured as undifferentiated “time” but become more intelligible once we attend to which adolescents are exposed to what kinds of experiences and to the processes that carry risk.

Across outcomes, associations are often stronger for girls, particularly where appearance-focused content, social comparison, and sleep disruption are involved. This asymmetry likely reflects both differential exposure, greater engagement with photo-centric feeds, and differential susceptibility during mid-adolescence, when identity work and peer evaluation intensify [37,38]. Age and developmental stage matter in their own right: several cohorts indicate that mid-adolescence is a period of heightened sensitivity, whereas patterns can attenuate later [37]. Baseline mental health is another consistent moderator. Adolescents who begin with elevated depressive or anxiety symptoms tend to show stronger adverse associations and, in bidirectional models, are more likely to increase problematic use over time, consistent with reverse-causation dynamics [16]. Individual differences help to explain why ostensibly similar online exposures have different consequences: neuroticism and rumination appear to amplify reactivity to negative or comparison-laden cues, though estimates vary across studies and designs [54] more reliably with worse momentary mood than active, social engagement, yet within-person effects are typically small and person-specific; similarly, photo-centric platforms yield more consistent links to body-image concerns than text-centric use [40,55,56,57]. Family context can buffer risk: higher parental monitoring and offline support attenuate adverse associations for some outcomes [58].

The emerging picture is one of heterogeneous risk: effects are more likely when susceptible adolescents (e.g., girls in mid-adolescence or youth with higher baseline symptoms or ruminative tendencies) engage in specific processes (appearance-focused, passive, or conflict-prone interactions) that activate mediators (sleep loss, harassment, comparison-related cognitions, or dysregulation). This clarifies why objective log-based or within-person studies that treat time as a single quantity often return null or very small effects, whereas content- and process-specific analyses can detect meaningful, if still modest, associations. Consistent with this, we avoid causal language for cross-sectional findings, give greater interpretive weight to longitudinal and daily-diary designs, and present null and reverse-causal results alongside positive findings. Table 2 summarizes key moderators and mediators influencing the relationship between social media use and adolescent mental health.

## 8. Implications

The evidence presented in this review highlights a complex, multifaceted relationship between social media use and adolescent mental health. Rather than being universally harmful or beneficial, social media acts as a context-sensitive amplifier of both risk and resilience factors, depending on who is using it, how, and under what circumstances. This nuanced understanding has significant implications for clinicians, educators, policymakers, parents, and young users themselves.

### 8.1. Beyond “Screen Time”: The Need for Precision

Traditional discourse on digital health has often focused on limiting overall “screen time.” However, this approach oversimplifies the issue and overlooks the quality, context, and content of digital interactions [60]. Research consistently shows that not all screen time is equal: scrolling through curated feeds passively is not equivalent to chatting with friends, seeking information, or engaging in creative expression. Future interventions should thus emphasize digital literacy and intentional use rather than time-based restrictions alone. Adolescents need support in developing healthy online habits, critically assessing content, and navigating peer interactions online with awareness and boundaries.

### 8.2. The Role of Families and Schools

Families play a pivotal role in shaping adolescents’ digital experiences. Parental monitoring, co-use, and open communication have been shown to mitigate the risks associated with harmful content and peer comparison [54]. Yet many parents feel underprepared to guide their children in this domain and may themselves lack the literacy to recognize harmful patterns of use. Educational institutions can act as key partners in promoting digital well-being. By integrating topics such as cyberbullying, self-image, and online empathy into the curriculum, schools can help normalize mental health discussions and equip students with tools for safe engagement. Schools can foster a supportive climate, create resources for students, staff, and families that address responsible technology use, online safety, and healthy digital habits.

### 8.3. Opportunities for Positive Engagement

It is important to acknowledge that social media is not inherently detrimental. For many adolescents, it provides a vital source of social connection, especially for those who feel marginalized or isolated offline. It allows youth to explore identity, participate in community-building, and access mental health resources that may not be available in their physical environment [61]. Indeed, emerging research suggests that under supportive conditions, social media use can promote self-expression, social belonging, and resilience [62]. The goal should, therefore, be to cultivate environments, both online and offline, that amplify protective factors and reduce the amplification of harm.

### 8.4. Toward Evidence-Based Guidelines and Platform Responsibility

At a policy level, stakeholders must move beyond moral panic and toward evidence-based digital health guidance. This includes funding longitudinal research, regulating algorithmic amplification of harmful content, and holding platforms accountable for the mental health consequences of their design features [63]. Tech companies should prioritize safety-by-design, such as time limit prompts, suicide prevention resources, AI-based flagging of risky behaviors or content, user control over feed customization, and comment moderation. Clinicians and researchers must also remain cautious of over-pathologizing normal behavior and instead differentiate between problematic use and adaptive engagement. Personalized assessments and culturally sensitive approaches will be critical in the years ahead.

**Figure 3:**
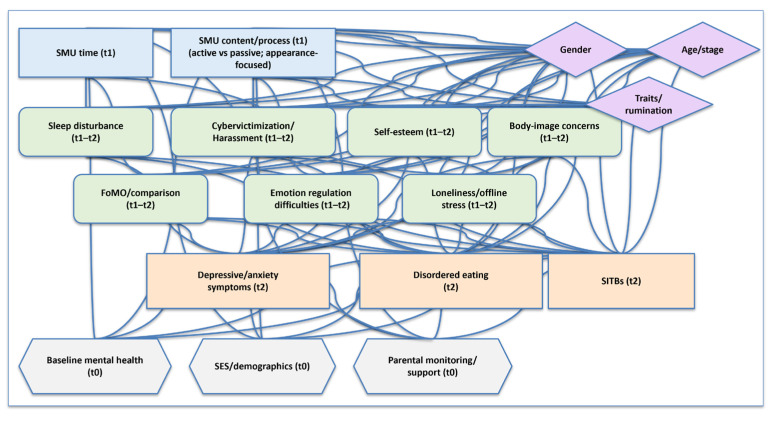
Directed acyclic graph (DAG) of hypothesized relations among social media use, mechanisms, and adolescent mental health. Legend: blue = exposures (t_1_); green = mediators (t_1_–t_2_); orange = outcomes (t_2_); gray hexagon = confounders (t_0_); purple diamonds = moderators (effect modifiers). Arrows indicate hypothesized causal directions with temporal ordering (t_0_ → t_1_ → t_2_). Blue nodes denote exposures at time t_1_ (total SMU time; content/process such as active vs. passive and appearance-focused engagement). Green nodes are mediators (t_1_–t_2_: sleep disturbance; cybervictimization/harassment; self-esteem; body-image concerns; emotion-regulation difficulties; FoMO/comparison; loneliness/offline stress). Orange nodes are outcomes at t_2_ (depressive/anxiety symptoms; disordered eating; SITBs). Gray hexagons are confounders measured at t_0_ (baseline mental health, SES/demographics, and parental monitoring/support) that may influence both exposure and outcomes. Purple diamonds indicate moderators/effect modifiers (gender, age/developmental stage, and personality traits/rumination) that may alter effect size or direction. Arrows represent hypothesized causal directions with temporal ordering (t_0_ → t_1_ → t_2_). This schematic clarifies indirect pathways (e.g., SMU → sleep loss → symptoms), potential reverse-causation captured as baseline mental health (t_0_) → later SMU (t_1_), and the distinction between confounders (to adjust for) and mediators (not to adjust for when estimating total effects). Abbreviations: DAG, directed acyclic graph; SMU, social media use; FoMO, fear of missing out; SITBs, self-injurious thoughts and behaviors; SES, socioeconomic status; t_0_/t_1_/t_2_, time points (baseline/exposure and mediators/outcomes); vs, versus.

To clarify our theoretical framework, Figure 3 depicts a DAG with exposures at t_1_ (SMU time; content/process), mediators at t_1_–t_2_ (sleep disturbance, cybervictimization, self-esteem, body-image concerns, emotion-regulation difficulties, FoMO/comparison, and loneliness/offline stress), and outcomes at t_2_ (depressive/anxiety symptoms, disordered eating, and SITBs). Confounders observed at t_0_ (baseline mental health, SES/demographics, and parental monitoring/support) are shown as common causes of exposures and outcomes; moderators (gender, age/stage, and traits/rumination) are depicted as effect modifiers. The DAG helps differentiate paths to adjust for (confounders) from indirect pathways (mediators) and illustrates how reverse-causation is handled via temporal indexing (baseline symptoms → later SMU). We draw on this schematic when interpreting heterogeneous findings and when prioritizing longitudinal/within-person designs for causal inference.

## 9. Discussion and Conclusions

Despite the growing body of research on the relationship between social media use and adolescent mental health, several limitations in the current literature must be acknowledged. Most existing studies are cross-sectional, limiting our ability to draw conclusions about causality or temporal dynamics. While some longitudinal and experimental designs have emerged, their results are often heterogeneous due to differing methodologies, definitions of “use,” and measurement of mental health outcomes [64]. Another challenge is the overreliance on self-report measures, which can introduce biases related to social desirability, recall error, and lack of insight, especially in younger populations. Furthermore, most studies do not account for platform-specific effects, despite significant differences in features, content algorithms, and user demographics between platforms such as Instagram, TikTok, Snapchat, and Discord. Cultural generalizability is also a concern: much of the available data comes from Western, industrialized countries. As a result, the role of socioeconomic status, cultural values, and family dynamics in shaping digital experiences remains underexplored. This limits the applicability of findings in non-Western settings or marginalized communities [65].

Overall, the average associations between generic social-media time and mental-health outcomes are small and sometimes null, particularly in designs using objective logs or within-person analyses. Where effects emerge, they are more consistent for content/process-specific exposures (e.g., appearance-focused engagement and cybervictimization) than for undifferentiated time. The field’s main limitations include heavy reliance on self-report for both exposure and outcomes, non-probability sampling, inconsistent operationalizations of “use,” and limited pre-registration.

In interpreting associations between SMU and youth mental health, we explicitly consider reverse causation, the possibility that adolescents with pre-existing difficulties are more likely to seek or sustain particular online activities. Conceptually and empirically, both pathways are plausible [16]. Bidirectional models and longitudinal evidence indicate that baseline depressive symptoms can prospectively predict increases in problematic SMU, whereas SMU does not consistently predict subsequent depressive symptoms when temporal ordering and covariates are accounted for. Consistent with this, a broader reverse-causation perspective argues that selection into certain online behaviors by already-vulnerable youth can explain part of the observed correlation between SMU and distress [15]. Taken together with the small or null average effects observed for generic time-based metrics, these findings suggest that directionality is heterogeneous and may depend on who is using (vulnerabilities), what they do online (content/process), and how outcomes are measured. Accordingly, it is pivotal to avoid causal language for cross-sectional results, foreground longitudinal/within-person evidence, and interpret associations cautiously.

Recent work shows clear strengths: growth of large longitudinal cohorts, rising use of daily-diary/ESM to isolate within-person dynamics, occasional access to objective usage logs, and greater attention to mediators (sleep, harassment, and self-esteem) and moderators (gender, age, and traits). At the same time, several limitations recur across the literature: self-report dependence for both exposure and outcome, inviting recall and common-method bias (“time on social media” is often a coarse proxy that omits content/context); design mix skewed to cross-sectional studies, constraining causal inference; even longitudinal work may not fully address reverse causation or time-varying confounding; variability in exposure definitions (screen time vs. platform-specific behaviors vs. problematic use), hindering synthesis and comparability; limited pre-registration/registered reports and heterogeneous analytic choices; selective reporting risk; sampling and generalizability issues (non-probability samples; WEIRD/country-specific cohorts; under-representation of non-Western contexts); small average effect sizes, requiring adequate power, precision, and cautious interpretation [66].

The current literature makes clear that when and how young people use social media matters as much as how much they use it. Moving beyond generic screen-time totals, future work should follow adolescents closely over time and within time, capturing day-to-day and even hour-to-hour fluctuations in mood, sleep, stress, and body image alongside what they actually do online. Micro-longitudinal daily-diary/experience-sampling designs are well suited to this task. By prompting youth several times per day across multi-week “bursts,” researchers can probe short lags (minutes/hours), examine non-linearities and thresholds (e.g., how much is too much under specific conditions), and test whether effects look different on school nights versus weekends, during exams, or after particular online experiences (e.g., prolonged exposure to appearance-focused feeds).

Equally important is establishing what our measures mean. Device-level “screen-time” counters and platform-level activity logs can diverge in ways that matter (multi-device switching, background activity, rounding of minutes, time-zone drift, and app misclassification). We, therefore, see a clear role for validation and audit studies: pre-registered protocols that compare different logging sources, quantify agreement, map missing-data patterns, and document how settings or updates alter what is recorded. A validated measurement layer will make findings more comparable across labs and platforms and will reduce the risk of over-interpreting noisy proxies [67].

Content and process deserve the same care. Rather than treating all time as equal, mixed-method designs can link objective logs to what was viewed or done (e.g., appearance-centric scrolling, social comparison cues, and cybervictimization exposure) and to how it was experienced. Privacy-preserving approaches, manual coding of small samples, user-curated screenshots, or lightweight NLP/computer-vision methods, can be paired with event-triggered surveys (e.g., a brief prompt after 15 min of photo-feed browsing). This pairing gives traction on mechanisms that simple duration metrics miss and helps explain why effects are small on average yet meaningful for specific subgroups or contexts.

Clarifying directionality will also require designs that tackle confounding head-on. Within-person and longitudinal models, such as random-intercept cross-lagged panel models, fixed-effects regression, marginal structural models with inverse-probability weighting, or case-crossover approaches, can separate stable between-person differences from genuine changes within the same adolescent. Used alongside daily-diary data, these tools can test both pathways (social media → later symptoms and symptoms → later social media patterns), helping to adjudicate reverse-causation accounts and to identify when associations are most likely to be causal [68].

Finally, mixed methods should be taken literally: pairing quantitative traces with qualitative diaries or interviews often reveals the offline context and intentions behind online behavior (seeking support, distraction before sleep, body-image checking, and coping after bullying). Triangulation across methods and data sources, including wearables for sleep, ambient light/noise sensors, or calendar context, can clarify mediator pathways with objective measures. Across all of these directions, greater transparency will strengthen the evidence base: pre-registration or registered reports, multiverse/specification-curve analyses, open codebooks for exposure categories, and cross-platform harmonization will make results more reproducible. Just as crucial is diversity: studies should prioritize cross-cultural samples and under-represented groups and report recruitment, attrition, and measurement invariance so readers can judge generalizability.

In short, the field’s next step is not simply “more longitudinal studies” but more precise, validated, and context-aware longitudinal and mixed-method designs that illuminate mechanisms, clarify directionality, and explain for whom and under what conditions social media relates to adolescent mental health.

Future studies should prioritize longitudinal and mixed-method designs to better capture the dynamic interplay between online behaviors and developmental trajectories. Incorporating ecological momentary assessments and passive digital data (e.g., screen logs and activity metrics) could reduce reliance on self-report and provide more granular insights. There is also a pressing need to investigate protective factors and resilience pathways. Most studies have focused on risks and harms, leaving a relative gap in our understanding of how positive digital experiences, such as peer support, identity exploration, or mental health advocacy, can promote psychological well-being [69]. Importantly, interdisciplinary collaborations between psychologists, data scientists, educators, and designers will be essential to create interventions that are developmentally appropriate, culturally sensitive, and technologically realistic. Social media is an inescapable feature of modern adolescence. It offers both opportunities and risks, amplifying individual vulnerabilities but also enabling connection, self-expression, and growth.

We encourage multi-source measurement (self-report + device logs + content coding of feeds/interactions); pre-registered analyses/registered reports, with robustness via multiverse/specification-curve; designs that prioritize within-person and temporal ordering (Random-Intercept Cross-Lagged Panel Model or RI-CLPM, fixed-effects, or marginal structural models/Inverse Probability of Treatment Weighting or IPTW), and where feasible natural experiments; a shared exposure taxonomy distinguishing time, activity type, content/valence, social context, and algorithmic curation; cross-cultural replication/harmonized outcomes with measurement invariance; routine reporting of effect sizes with 95% CIs, open data/code, and bias-sensitive sensitivity analyses. Additionally, we recommend integrating qualitative and participatory components into future studies and program design: brief post-event interviews linked to logs/ESM, youth-led audits of platform experiences, co-designed school toolkits on sleep-aware use and appearance-focused content, and scenario-based workshops for educators to rehearse responses to cybervictimization or comparison spirals. Such outputs translate research into concrete guidance (checklists, classroom activities, and referral pathways) that can be adapted across cultural contexts.

The evidence reviewed in this paper underscores that the impact of social media on mental health is neither inherently harmful nor uniformly benign but can be considered conditional, context-dependent, and deeply influenced by individual, social, and environmental factors.

Rather than demonizing digital platforms or prescribing one-size-fits-all guidelines, researchers and stakeholders must focus on differentiated, evidence-based strategies that empower adolescents to engage with social media safely, consciously, and creatively. Supporting digital well-being is not about eliminating screens but about teaching young people to thrive with them.

## 10. Limitations

This review should be interpreted in light of several limitations of the underlying evidence base. Most available studies are cross-sectional, which constrains causal inference and temporal precedence; even longitudinal work may not fully address reverse causation (symptoms → later patterns of use) or time-varying confounding. The majority of studies rely on self-reported social media use and mental-health outcomes, raising concerns about recall error, social desirability, and common-method bias. While a subset uses objective logs, their coverage and validity vary across devices/platforms (e.g., multi-device use, background activity, and rounding), and generic time-based metrics are often a coarse proxy for risk-relevant experiences (content/process). There is substantial measurement heterogeneity: constructs such as “problematic use,” “internet addiction,” and “compulsive use” are operationalized with different instruments and thresholds; exposure definitions (time vs. activity type vs. content) and outcome scales vary, complicating synthesis and comparability. Typical effect sizes are small and sometimes null, and many studies lack pre-registration/registered reports or multiverse/specification-curve analyses, leaving room for analytic flexibility and selective reporting. Fifth, samples are frequently non-probabilistic and concentrated in WEIRD contexts, with limited coverage of non-Western platforms, socioeconomic diversity, and cultural variation in digital ecologies, constraining generalizability. Finally, specialized subfields (e.g., neuroimaging) often involve small samples and task heterogeneity, limiting power and reproducibility. Together, these constraints counsel cautious interpretation of associations, emphasize the need for design-sensitive weighting of evidence (especially within-person and longitudinal designs with validated measures), and motivate future work that integrates objective logging, content-aware measurement, pre-registered analyses, and cross-cultural sampling.

## Figures and Tables

**Table 1:** Key associations between social media use and adolescent mental health outcomes.

Claim/Construct	Association Type	Representative Reference(s)	Study Design/Sample	Effect-Size Metric	Point Estimate (95% CI)	Notes
Digital technology use vs. adolescent well-being (magnitude)	Correlational (cross-sectional; specification-curve)	Orben and Przybylski, 2019 [36]	SCA across 3 large national datasets; adolescents 12–18 y; N_tot = 355,358 Design: XS + SCA; Measure: SR; N-band: VL (≥10,000)	R^2^ (variance explained)	R^2^ ≤ 0.004 (≤0.4% of variance); CI: NR	Negative but trivially small association across specifications.
Daily social media use intensity and depressive symptoms (gender differences)	Association gradient; mediation paths	Kelly et al., 2019 [37]	UK Millennium Cohort Study; cross-sectional path analysis; n = 10,904; age ≈ 14 Design: XS (path); Measure: SR; N-band: VL (≥10,000)	% change in depressive symptom score (vs. 1–3 h/day reference)	Girls: 3–<5 h = +26%; ≥5 h = +50% Boys: 3–<5 h = +21%; ≥5 h = +35% Body dissatisfaction → depressive symptoms: +15% ≥5 h → body dissatisfaction: +31% more likely (CIs not reported)	Multiple pathways via sleep, online harassment, self-esteem, body image.
Problematic social media use and poor sleep health indicators	Adjusted odds (cross-sectional)	Lafontaine-Poissant et al., 2024 [38]	HBSC Canada 2017–2018; mixed-effects logistic models; n = 12,557 (11–17 y) Design: XS; Measure: SR; N-band: VL (≥10,000)	aOR	Problematic vs. active SMU: aORs 1.67–3.24 across 7 indicators. Examples: Insomnia symptoms aOR = 3.24 (2.61–4.02); Daytime wakefulness aOR = 2.67 (2.15–3.31); Screen time before bed aOR = 2.76 (1.86–4.08); Not meeting sleep duration recs aOR = 2.43 (2.01–2.93); Sleep variability aOR = 2.23 (1.79–2.77); Late bedtime (school days) aOR = 2.89 (2.20–3.79).	Associations stronger in girls (e.g., late bedtime: girls aOR 3.74 vs. boys 1.84; non-school days 4.13 vs. 2.18).
Social media use and suicidal feelings (adolescents)	Meta-analytic odds	Macrynikola et al., 2021 [39]	Systematic review/meta-analysis Design: MA/SR; Measure: SR; N-band: varies	OR	OR = 1.36 (1.10–1.67) for suicidal feelings among adolescents	Cross-sectional evidence predominates; calls for more longitudinal research.
Social media use and body image disturbance	Meta-analytic correlation	Saiphoo and Vahedi, 2019 [40]	Meta-analysis across published studies Design: MA/SR; Measure: SR; N-band: varies	r	r = 0.156 (95% CI 0.123–0.188)	Effects stronger for appearance-focused/photo-based use than general use; small overall magnitude.
Reverse causation: depressive symptoms → later SMU; SMU ↛ later depressive symptoms	Bidirectional (cross-lagged panel)	Heffer et al., 2019 [16]	Adolescents (n = 597; 2 waves) and young adults (n = 1132; 6 waves) Design: CLP; Measure: SR; N-band: M–L (≈600 and 1132)	Standardized β (cross-lag)	Adolescent females: Dep_1_ → SM_2_ β = 0.131 (0.026–0.236); SM_1_ → Dep_2_ β = −0.043 (−0.159–0.073), ns. Adolescent males: SM_1_ → Dep_2_ β = 0.145 (0.000–0.288), *p* ≈ 0.051 (ns with covariates); Dep_1_ → SM_2_ β = 0.094 (−0.033–0.220), ns.	Supports depressive symptoms predicting later SMU in girls; minimal evidence for SMU → later depression.

Notes and abbreviations: SMU = social media use; SCA = specification curve analysis; aOR = adjusted odds ratio; OR = odds ratio; r = Pearson correlation; β = standardized regression coefficient; CI = 95% confidence interval; NR = not reported. Kelly et al. [37] report percentage changes in depressive symptom scores relative to a 1–3 h/day reference group; exact CIs for those percentage changes are not provided in the main text. Problematic SMU in Lafontaine-Poissant et al. [38] was assessed with the Social Media Disorder Scale. Heffer et al. [16] coefficients shown here are from models including covariates; see article tables for full models. Construct labels follow the instrument used: PSMU (BSMAS/SMD); PIU (IAT); CU (CIUS); IGD refers to gaming disorder only. Quality flags—design: XS = cross-sectional; L = longitudinal; CLP = cross-lagged panel; MA/SR = meta-analysis/systematic review. Measure: SR = self-report; OBJ = objective logs. Sample size bands: S < 200; M 200–999; L ≥ 1000; VL ≥ 10,000.

**Table 2:** Moderators and mediators of associations between social media use and adolescent mental health.

Type	Factor	Operationalization/Example Measure	Expected Direction (Summary)	Study Design/Measure	Representative Evidence	Notes
Mediator	Sleep disturbance	Sleep duration/quality; insomnia symptoms; bedtime variability	Higher SMU → poorer sleep → higher depressive/anxiety symptoms	L; SR (some OBJ time)	Kelly et al., 2019 [37]	Gender-differentiated pathways reported in UK cohort; also supported by reviews.
Mediator	Cybervictimization/online harassment	Self-report frequency in past 6–12 months	Higher SMU → ↑ cybervictimization → ↑ depressive/suicidal symptoms	L/XS; SR	Kelly et al., 2019 [37]; Macrynikola et al., 2021 [39]	Often co-occurs with offline bullying; temporality clearer in cohorts.
Mediator	Self-esteem	Rosenberg Self-Esteem Scale	Higher SMU (esp. passive/appearance-focused) → ↓ self-esteem → ↑ depressive symptoms	L/XS; SR	Kelly et al., 2019 [37]; Saiphoo and Vahedi, 2019 [40]	Effects small on average; content/process specificity matters.
Mediator	Body image concerns	SATAQ; body dissatisfaction scales	Appearance-focused activities → ↑ body concern → ↑ disordered eating/depression	MA/XS; SR	Saiphoo and Vahedi, 2019 [40]; Holland et al., 2016 [59]	Larger effects for photo-based SNS; heterogeneity across measures.
Mediator	Emotion regulation difficulties	DERS; dysregulation indices	Problematic/compulsive use → ↑ emotion dysregulation → ↑ symptoms	XS/L; SR	Heffer et al., 2019 [16]	Directionality mixed; see CLP evidence for reverse paths as well.
Mediator	FoMO/upward social comparison	FoMO scale; comparison orientation scales	Passive/scrolling/photo activities → ↑ FoMO/comparison → ↑ distress	XS/DD; SR	Holland et al., 2016 [59]; Orben et al., 2019 [36]	Mechanistic plausibility; average effects small.
Mediator	Loneliness/offline stress	UCLA loneliness; life event checklists	SMU under stress → ↑ loneliness/stress → ↑ symptoms	L/XS; SR	Boers et al., 2019 [41]	Time-varying confounding possible; consider within-person designs.
Moderator	Gender	Male/female; gender identity where available	Stronger associations for girls in several outcomes (sleep, body image, depression)	L/XS; SR	Kelly et al., 2019 [37]; Lafontaine-Poissant et al., 2024 [38]	See sleep health and body-image pathways; not universal across outcomes.
Moderator	Developmental stage/age	Early vs. mid vs. late adolescence	Effects vary by age; mid-adolescence often more sensitive	L/XS; SR	Kelly et al., 2019 [37]	Developmental transitions and peer salience may amplify effects.
Moderator	Baseline mental health/vulnerability	Baseline depression/anxiety; clinical subgroup	Higher baseline symptoms → stronger SMU–outcome associations	CLP/L; SR	Heffer et al., 2019 [16]; Hartanto et al., 2021 [15]	Reverse-causation pathway: symptoms → later problematic SMU.
Moderator	Personality traits (e.g., neuroticism)/rumination	BFI/EPQ; RRS rumination scale	Higher neuroticism/rumination → stronger adverse associations	XS; SR	Odgers and Jensen, 2020 [54]	Theoretical synthesis; empirical evidence heterogeneous.
Moderator	Active vs. passive use	Posting/messaging vs. browsing/scrolling ratios	Passive use often linked to worse mood; active engagement less so	XS/DD; SR/OBJ	Valkenburg et al., 2022 [55]; Beyens et al., 2020 [56]	Within-person effects small/variable; content still key.
Moderator	Platform/content type (photo-based)	Instagram/photo-centric vs. text-centric activities	Photo-based use → stronger body-image links	MA/XS; SR	Saiphoo and Vahedi, 2019 [40]; Ryding and Kuss, 2020 [57]	Appearance-focused content amplifies risk pathways.
Moderator	Parental monitoring/offline support	Parenting practices; perceived support	Higher monitoring/support → weaker adverse associations (buffer)	XS/L; SR	Nesi et al., 2021 [58]	Buffering effects vary; more causal evidence needed.

Notes. Entries are representative and emphasize studies with clearer temporal ordering, within-person designs, meta-analytic summaries, or large samples where available. “Expected direction” summarizes typical patterns; effects are often small and heterogeneous across subgroups and measures. Abbreviations: BFI, Big Five Inventory; CLP: cross-lagged panel; DD, daily-diary; DERS, Difficulties in Emotion Regulation Scale; EPQ, Eysenck Personality Questionnaire; FoMO, fear of missing out; L, longitudinal (cohort); MA, meta-analysis; OBJ, objective logs (device/platform data); RRS, Ruminative Responses Scale; SATAQ, Sociocultural Attitudes Toward Appearance Questionnaire; SMU, social media use; SNS, social networking sites; SR, self-report; XS, cross-sectional.

## Data Availability

No new data were created for this study.

## References

[1] Keles B., McCrae N., Grealish A. (2020). A systematic review: The influence of social media on depression, anxiety and psychological distress in adolescents. Int. J. Adolesc. Youth.

[2] Twenge J.M., Martin G.N. (2020). Gender differences in associations between digital media use and psychological well-being: Evidence from three large datasets. J. Adolesc..

[3] Nesi J., Telzer E.H., Prinstein M.J. (2021). Adolescent developmental pathways to risk for suicide: Integrating brain and behavioral science to inform prevention. Psychol. Rev..

[4] Casey B.J., Jones R.M., Hare T.A. (2008). The adolescent brain. Ann. N. Y. Acad. Sci..

[5] Luna B., Marek S., Larsen B., Tervo-Clemmens B., Chahal R. (2015). An integrative model of the maturation of cognitive control. Annu. Rev. Neurosci..

[6] Fardouly J., Vartanian L.R. (2016). Social media and body image concerns: Current research and future directions. Curr. Opin. Psychol..

[7] Czubaj N., Szymańska M., Nowak B., Grajek M. (2025). The Impact of Social Media on Body Image Perception in Young People. Nutrients.

[8] Vannucci A., Flannery K.M., Ohannessian C.M. (2017). Social media use and anxiety in emerging adults. J. Affect. Disord..

[9] George M.J., Odgers C.L. (2015). Seven fears and the science of how mobile technologies may be influencing adolescents in the digital age. Perspect. Psychol. Sci..

[10] Montag C., Walla P. (2016). Carpe diem instead of losing your social mind: Beyond digital addiction and why we all suffer from digital overuse. Cogent Psychol..

[11] Verduyn P., Ybarra O., Résibois M., Jonides J., Kross E. (2017). Do social network sites enhance or undermine subjective well-being? A critical review. Soc. Issues Policy Rev..

[12] Brand M., Young K.S., Laier C. (2016). Prefrontal control and internet addiction: A theoretical model and review of neuropsychological and neuroimaging findings. Front. Hum. Neurosci..

[13] Festinger L. (1954). A theory of social comparison processes. Hum. Relat..

[14] Wrzus C., Neubauer A.B. (2023). Ecological Momentary Assessment: A Meta-Analysis on Designs, Samples, and Compliance Across Research Fields. Assessment.

[15] Hartanto A., Quek F.Y.X., Tng G.Y.Q., Yong J.C. (2021). Does social media use increase depressive symptoms? A reverse causation perspective. Front. Psychiatry.

[16] Heffer T., Good M., Daly O., MacDonell E.T., Willoughby T. (2019). The longitudinal association between social-media use and depressive symptoms among adolescents and young adults: An empirical reply to Twenge et al. (2018). Clin. Psychol. Sci..

[17] Shin N.Y. (2022). Psychometric properties of the Bergen Social Media Addiction Scale in Korean young adults. Psychiatry Investig..

[18] Boer M., Stevens G.W.J.M., Finkenauer C., Koning I.M., van den Eijnden R.J.J.M. (2022). Validation of the Social Media Disorder Scale in adolescents: Findings from a large-scale nationally representative sample. Assessment.

[19] Meerkerk G.J., van den Eijnden R.J., Vermulst A.A., Garretsen H.F. (2009). The Compulsive Internet Use Scale (CIUS): Some psychometric properties. Cyberpsychol. Behav..

[20] Darvesh N., Radhakrishnan A., Lachance C.C., Nincic V., Sharpe J.P., Ghassemi M., Straus S.E., Tricco A.C. (2020). Exploring the prevalence of gaming disorder and Internet gaming disorder: A rapid scoping review. Syst. Rev..

[21] Meshi D., Morawetz C., Heekeren H.R. (2013). Nucleus accumbens response to gains in reputation for the self relative to gains for others predicts social media use. Front. Hum. Neurosci..

[22] Nobakht H.N., Wichstrøm L., Steinsbekk S. (2025). Longitudinal Relations Between Social Media Use and Cyberbullying Victimization Across Adolescence: Within-Person Effects in a Birth Cohort. J. Youth Adolesc..

[23] Guyer A.E., Silk J.S., Nelson E.E. (2016). The neurobiology of the emotional adolescent: From the inside out. Neurosci. Biobehav. Rev..

[24] Crone E.A., Konijn E.A. (2018). Media use and brain development during adolescence. Nat. Commun..

[25] Dong N., Zhou Y., Lei L., Lee T.M.C., Lam C.L.M. (2025). The longitudinal impact of screen media activities on brain function, architecture and mental health in early adolescence. Int. J. Clin. Health Psychol..

[26] Şimşek O.M., Gümüşçü N., Koçak O., Alkhulayfi A.M.A., Gómez-Salgado J., Yıldırım M. (2025). Learning and performance orientation, life satisfaction and problematic social media use in high school and university students: A moderated mediation. Acta Psychol..

[27] Fardouly J., Diedrichs P.C., Vartanian L.R., Halliwell E. (2015). Social comparisons on social media: The impact of Facebook on young women’s body image concerns and mood. Body Image.

[28] Twenge J.M., Campbell W.K. (2018). Associations between screen time and lower psychological well-being among children and adolescents: Evidence from a population-based study. Prev. Med. Rep..

[29] Orben A., Przybylski A.K. (2019). Screens, teens, and psychological well-being: Evidence from three time-use-diary studies. Psychol. Sci..

[30] Marano G., Anesini M.B., Milintenda M., Acanfora M., Calderoni C., Bardi F., Lisci F.M., Brisi C., Traversi G., Mazza O. (2025). Neuroimaging and Emotional Development in the Pediatric Population: Understanding the Link Between the Brain, Emotions, and Behavior. Pediatr. Rep..

[31] Tonioni F., Mazza M., Autullo G., Pellicano G.R., Aceto P., Catalano V., Marano G., Corvino S., Martinelli D., Fiumana V. (2018). Socio-emotional ability, temperament and coping strategies associated with different use of Internet in Internet addiction. Eur. Rev. Med. Pharmacol. Sci..

[32] Lai C., Altavilla D., Mazza M., Scappaticci S., Tambelli R., Aceto P., Luciani M., Corvino S., Martinelli D., Alimonti F. (2017). Neural correlate of Internet use in patients undergoing psychological treatment for Internet addiction. J. Ment. Health.

[33] Tonioni F., Mazza M., Autullo G., Cappelluti R., Catalano V., Marano G., Fiumana V., Moschetti C., Alimonti F., Luciani M. (2014). Is Internet addiction a psychopathological condition distinct from pathological gambling?. Addict. Behav..

[34] Orben A., Przybylski A.K. (2019). The association between adolescent well-being and digital technology use. Nat. Hum. Behav..

[35] Goh A.Y.H., Hartanto A., Kasturiratna K.T.A.S., Majeed N.M. (2025). No consistent evidence for between- and within-person associations between objective social media screen time and body image dissatisfaction: Insights from a daily diary study. Soc. Media + Soc..

[36] Orben A., Dienlin T., Przybylski A.K. (2019). Social media’s enduring effect on adolescent life satisfaction. Proc. Natl. Acad. Sci. USA.

[37] Kelly Y., Zilanawala A., Booker C., Sacker A. (2019). Social media use and adolescent mental health: Findings from the UK Millennium Cohort Study. EClinicalMedicine.

[38] Lafontaine-Poissant F., Lang J.J., McKinnon B., Simard I., Roberts K.C., Wong S.L., Chaput J.-P., Janssen I., Boniel-Nissim M., Gariépy G. (2024). Social media use and sleep health among adolescents in Canada. Health Promot. Chronic Dis. Prev. Can..

[39] Macrynikola N., Auad E., Menjivar J., Miranda R. (2021). Does social media use confer suicide risk? A systematic review of the evidence. Comput. Hum. Behav. Rep..

[40] Saiphoo A.N., Vahedi Z. (2019). A meta-analytic review of the relationship between social media use and body image disturbance. Comput. Hum. Behav..

[41] Boers E., Afzali M.H., Newton N., Conrod P. (2019). Association of screen time and depression in adolescence. JAMA Pediatr..

[42] Masri-Zada T., Martirosyan S., Abdou A., Barbar R., Kades S., Makki H., Haley G., Agrawal D.K. (2025). The Impact of Social Media & Technology on Child and Adolescent Mental Health. J. Psychiatry Psychiatr. Disord..

[43] So W.W.Y., Woo B.P.Y., Wong C., Yip P.S.F. (2023). Gender differences in the relationships between meaning in life, mental health status and digital media use during Covid-19. BMC Public Health.

[44] Kilby R., Mickelson K.D. (2025). Combating weight-stigmatization in online spaces: The impacts of body neutral, body positive, and weight-stigmatizing TikTok content on body image and mood. Front. Psychiatry.

[45] van Dalen M., Dierckx B., Pasmans S.G.M.A., Aendekerk E.W.C., Mathijssen I.M.J., Koudstaal M.J., Timman R., Williamson H., Hillegers M.H.J., Utens E.M.W.J. (2020). Anxiety and depression in adolescents with a visible difference: A systematic review and meta-analysis. Body Image.

[46] Mingoia J., Hutchinson A.D., Wilson C., Gleaves D.H. (2017). The relationship between social networking site use and the internalization of a thin ideal in females: A meta-analytic review. Front. Psychol..

[47] Mazzeo S.E., Weinstock M., Vashro T.N., Henning T., Derrigo K. (2024). Mitigating harms of social media for adolescent body image and eating disorders: A review. Psychol. Res. Behav. Manag..

[48] Shoib S., Chandradasa M., Nahidi M., Amanda T.W., Khan S., Saeed F., Swed S., Mazza M., Di Nicola M., Martinotti G. (2022). Facebook and Suicidal Behaviour: User Experiences of Suicide Notes, Live-Streaming, Grieving and Preventive Strategies-A Scoping Review. Int. J. Environ. Res. Public Health.

[49] Balt E., Mérelle S., Robinson J., Popma A., Creemers D., van den Brand I., van Bergen D., Rasing S., Mulder W., Gilissen R. (2023). Social media use of adolescents who died by suicide: Lessons from a psychological autopsy study. Child Adolesc. Psychiatry Ment. Health.

[50] Marcos V., Fariña F., Isorna M., López-Roel S., Rolán K. (2025). Problematic Use of the Internet and Cybervictimization: An Empirical Study with Spanish Adolescents. Behav. Sci..

[51] Andreassen C.S., Pallesen S., Griffiths M.D. (2017). The relationship between addictive use of social media, narcissism, and self-esteem: Findings from a large national survey. Addict. Behav..

[52] Coyne S.M., Rogers A.A., Zurcher J.D., Stockdale L., Booth M. (2020). Does time spent using social media impact mental health?: An eight-year longitudinal study. Comput. Hum. Behav..

[53] Chhabra J., Pilkington V., Benakovic R., Wilson M.J., La Sala L., Seidler Z. (2025). Social Media and Youth Mental Health: Scoping Review of Platform and Policy Recommendations. J. Med. Internet Res..

[54] Odgers C.L., Jensen M.R. (2020). Annual Research Review: Adolescent mental health in the digital age: Facts, fears, and future directions. J. Child Psychol. Psychiatry.

[55] Valkenburg P.M., Meier A., Beyens I. (2022). Social media use and well-being: What we know and what we need to know. Curr. Opin. Psychol..

[56] Beyens I., Pouwels J.L., van Driel I.I., Keijsers L., Valkenburg P.M. (2020). The effect of social media on well-being differs from adolescent to adolescent. Sci. Rep..

[57] Ryding F.C., Kuss D.J. (2020). The use of social networking sites, body image dissatisfaction, and body dysmorphic disorder: A systematic review of psychological research. Psychol. Pop. Media.

[58] Nesi J., Burke T.A., Bettis A.H., Kudinova A.Y., Liu R.T. (2021). Social media use and self-injurious thoughts and behaviors: A systematic review and meta-analysis. Clin. Psychol. Rev..

[59] Holland G., Tiggemann M. (2016). A systematic review of the impact of the use of social networking sites on body image and disordered eating outcomes. Body Image.

[60] Uhls Y.T., Ellison N.B., Subrahmanyam K. (2017). Benefits and costs of social media in adolescence. Pediatrics.

[61] Nesi J. (2020). The impact of social media on youth mental health: Challenges and opportunities. North Carol. Med. J..

[62] Best P., Manktelow R., Taylor B. (2014). Online communication, social media and adolescent wellbeing: A systematic narrative review. Child. Youth Serv. Rev..

[63] Nesi J., Telzer E.H., Prinstein M.J. (2020). Adolescent Development in the Digital Media Context. Psychol Inq..

[64] Rideout V., Fox S. (2018). Digital Health Practices, Social Media Use, and Mental Well-Being Among Teens and Young Adults in the U.S. Hopelab & Well Being Trust Report. https://hopelab.org/report/a-national-survey-by-hopelab-and-well-being-trust-2018/.

[65] McNamara P., Dunn N. (2025). Netflix’s Adolescence: An unsettling reminder of the toxic effect of social media on children. Br. J. Gen. Pract..

[66] Weng C.A., Bulgin J., Diaz S., Zhang J., Tan R., Li L., Armstrong-Hough M. (2025). Communication attributes modify the anxiety risk associated with problematic social media use: Evidence from a prospective diary method study. Addict. Behav..

[67] Osborne A. (2025). Balancing the benefits and risks of social media on adolescent mental health in a post-pandemic world. Child Adolesc. Psychiatry Ment. Health.

[68] Leung L. (2014). Predicting Internet risks: A longitudinal panel study of gratifications-sought, Internet addiction symptoms, and social media use among children and adolescents. Health Psychol. Behav. Med..

[69] Zimmermann E., Tomczyk S. (2025). The Ways of Using Social Media for Health Promotion Among Adolescents: Qualitative Interview and Focus Group Study. J. Med. Internet Res..

